# Screening of five marine-derived fungal strains for their potential to produce oxidases with laccase activities suitable for biotechnological applications

**DOI:** 10.1186/s12896-020-00617-y

**Published:** 2020-05-12

**Authors:** Wissal Ben Ali, Delphine Chaduli, David Navarro, Christian Lechat, Annick Turbé-Doan, Emmanuel Bertrand, Craig B. Faulds, Giuliano Sciara, Laurence Lesage-Meessen, Eric Record, Tahar Mechichi

**Affiliations:** 1 Ecole Nationale d’Ingénieurs de Sfax, Laboratoire de Biochimie et de Génie enzymatique des lipases, Université de Sfax, Sfax, Tunisie; 2 Biodiversité et Biotechnologie Fongiques, Aix-Marseille Université, INRA UMR1163, Marseille, France; 3 INRA, Aix-Marseille Université, UMR1163, CIRM-CF, Marseille, France; 4 Ascofrance, 64 route de Chizé, F-79360 Villiers-en-Bois, France

**Keywords:** Marine-derived fungi, *Trichoderma asperellum*, Laccase-like activity, Laccase, Dyes

## Abstract

**Background:**

Environmental pollution is one of the major problems that the world is facing today. Several approaches have been taken, from physical and chemical methods to biotechnological strategies (e.g. the use of oxidoreductases). Oxidative enzymes from microorganisms offer eco-friendly, cost–effective processes amenable to biotechnological applications, such as in industrial dye decolorization. The aim of this study was to screen marine-derived fungal strains isolated from three coastal areas in Tunisia to identify laccase-like activities, and to produce and characterize active cell-free supernatants of interest for dye decolorization.

**Results:**

Following the screening of 20 fungal strains isolated from the harbors of Sfax and Monastir (Tunisia), five strains were identified that displayed laccase-like activities. Molecular-based taxonomic approaches identified these strains as belonging to the species *Trichoderma asperellum*, *Stemphylium lucomagnoense* and *Aspergillus nidulans*. Among these five isolates, one *T. asperellum* strain (*T. asperellum* 1) gave the highest level of secreted oxidative activities, and so was chosen for further studies. Optimization of the growth medium for liquid cultures was first undertaken to improve the level of laccase-like activity in culture supernatants. Finally, the culture supernatant of *T. asperellum* 1 decolorized different synthetic dyes belonging to diverse dye families, in the presence or absence of 1-hydroxybenzotriazole (HBT) as a mediator.

**Conclusions:**

The optimal growth conditions to produce laccase-like active cell-free supernatants from *T. asperellum* 1 were 1.8 mM CuSO_4_ as an inducer, 1% NaCl to mimic a seawater environment and 3% sucrose as a carbon source. The culture supernatant of *T. asperellum* 1 effectively decolorized different synthetic dyes belonging to diverse chemical classes, and the presence of HBT as a mediator improved the decolorization process.

## Background

Water pollution is a serious environmental issue. Many industries are reported to dump wastes into rivers, lakes, ponds and streams to hide them from Environmental Protection Agencies [1]. Many studies have thus focused on microbial enzyme transformation and detoxification of pollutants [2, 3]. For this purpose, fungi are considered more robust than bacteria and are generally more tolerant to high concentrations of pollutants [4]. They produce high levels of extracellular enzymes with large industrial potential in eco-friendly, cost-effective processes [4].

Most fungi studied today are isolated from forests and other terrestrial environments. Few studies have explored marine fungal diversity [5]. Yet marine environments are extremely complex and host a broad spectrum of fungal species [6]. Although some novel fungal genera have been identified in marine environments and characterized, most marine-derived fungi seem to be related to terrestrial fungi, such as *Fusarium* sp., *Aspergillus* sp. and *Penicillium* sp. Marine-derived fungi have been shown to be present in various habitats, such as coastal areas, marine sediments and deep sea, associated with sponges, microalgae, fish and mangrove wood. Marine fungi have been classified as either obligate or facultative: obligate marine fungi grow exclusively in a marine habitat, whereas facultative marine fungi are of freshwater or terrestrial origin but are able to thrive in marine environments [7–9]. “The term marine-derived fungi is often used because most fungi isolated from marine samples are not demonstrably classified as obligate or facultative marine microorganisms” as described by Osterhage [10]. Recently, an online database was created to obtain more insight into the taxonomy of marine-derived fungi (www.marinefungi.org), with a full description of all known marine fungal species [11]. The utility of discovering the biodiversity of marine-derived fungi is not merely taxonomic: within each marine habitat, local microbial communities have adapted to seawater environmental conditions, and their enzymes are therefore potentially very attractive for biotechnology applications, owing to their properties, including thermostability, and salt and pH tolerance. Given their adaption to low temperature, high salinity, high pressure and oligotrophic conditions typical of the marine environment, marine-derived fungi are clearly a promising source of novel bioactive metabolites not found in terrestrial strains of the same species, including enzymes and laccases [9].

The laccases (EC 1.10.3.2) are a multigenic family of multicopper oxidases distributed across bacteria, fungi and plants. They catalyze, at a mononuclear copper center T1, the one-electron oxidation of four substrate molecules including substituted phenols, arylamines and aromatic thiols, to the corresponding radicals, with the simultaneous reduction at a trinuclear copper center T2/T3 of molecular oxygen to water [12]. The laccases form a large group of oxidoreductases, with a broad spectrum of substrates [12]. With their active copper cluster, they do not need any heterogeneously added cofactors for their activity, and their co-substrate, oxygen, is usually present in their environment. Most of these enzymes are naturally secreted and so are generally highly stable in the extracellular environment. The high level of inducible expression of laccase-encoding genes in most fungal species adds to their attractiveness in biotechnological applications [3]. New sources of laccases with special properties, such as high-redox potential, high salt and temperature tolerance, or cold adaptivity, are wanted for industrial applications. A broad variety of fungal strains isolated from several sea grasses, algae and decaying wood samples are able to produce laccases [13]. Mabrouk et al. [14] have isolated *Trematosphaeria mangrovei* from a mangrove ecosystem, which produces a laccase in significant quantities. A thermostable, metal-tolerant laccase is produced by the marine-derived fungus *Cerrena unicolor* [15]. Several researchers have isolated laccase-producing fungi from different sources, notably among the species *Trichoderma harzianum, Trichoderma atroviride, Trichoderma longibrachiatum*, *Trametes versicolor*, *Lentinus tigrinus, Trametes pubescens*, *Cyathus bulleri, Paecilomyces* sp., *Phanerochaete chrysosporium*, *Lentines edodes, Pleurotus ostreatus*, *Ganoderma lucidum, Alternaria tenuissima* and *Trichoderma* sp. [13]. Because fungi from marine environments have adapted to grow under high saline (15–34 ppt (parts per thousand)) and alkaline conditions, the laccases they produce are of potential interest for the bioremediation of high-salt and alkaline effluents, such as those from the pulp and paper, tanning and textile industries [16].

Reports on the identification of marine-derived laccases are still scant. The main purpose of this study was to isolate and identify new marine-derived fungal strains, to screen them for their capacity to produce laccase-active cell-free supernatants, and to determine, for a few selected strains, the optimal growth conditions for obtaining high levels of laccase-like activities.

## Results

### Isolation and identification of fungal strains

Marine-derived fungi from various marine areas of the Tunisian coast were isolated and screened. Twenty fungal strains were isolated up to the stage of monomorphic cultures in solid medium. Five of them showed positive oxidative activity on both DMP and ABTS added as substrates to solid medium in Petri dishes.

**Fig. 1:**
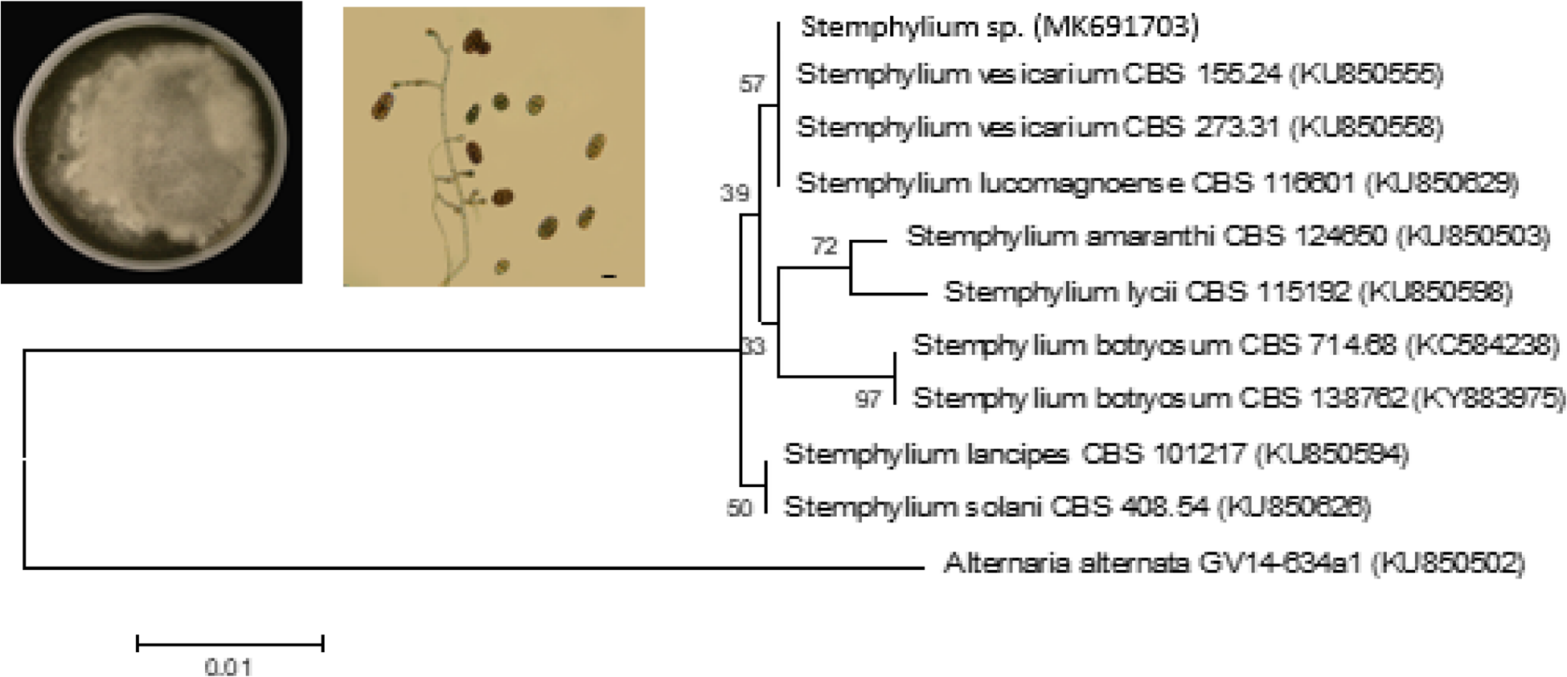
Phylogenetic reconstruction for the strain *Stemphylium* sp.*,* based on ITS analysis [17] using the neighbor-joining algorithm (NJ) method and 1000 replicate bootstraps. ITS sequences were deposited in the NCBI under accession number MK691703. Culture plate of *Stemphylium sp*. and conidiophores and conidia (scale bar = 10 μm)

**Fig. 2:**
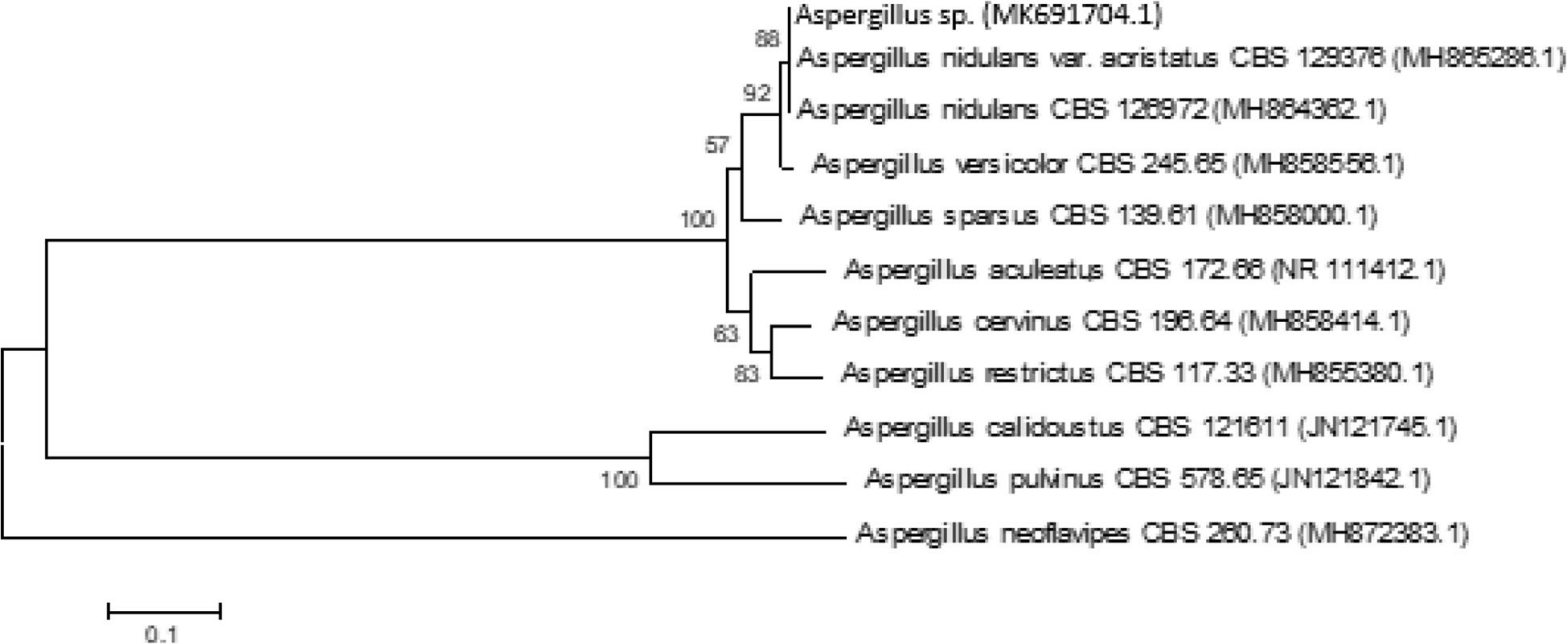
Phylogenetic reconstruction for the strain *Aspergillus sp,* based on ITS analysis using the neighbor-joining algorithm (NJ) method and 1000 replicate bootstraps, ITS sequences were deposited in the NCBI under accession number MK691704

**Fig. 3:**
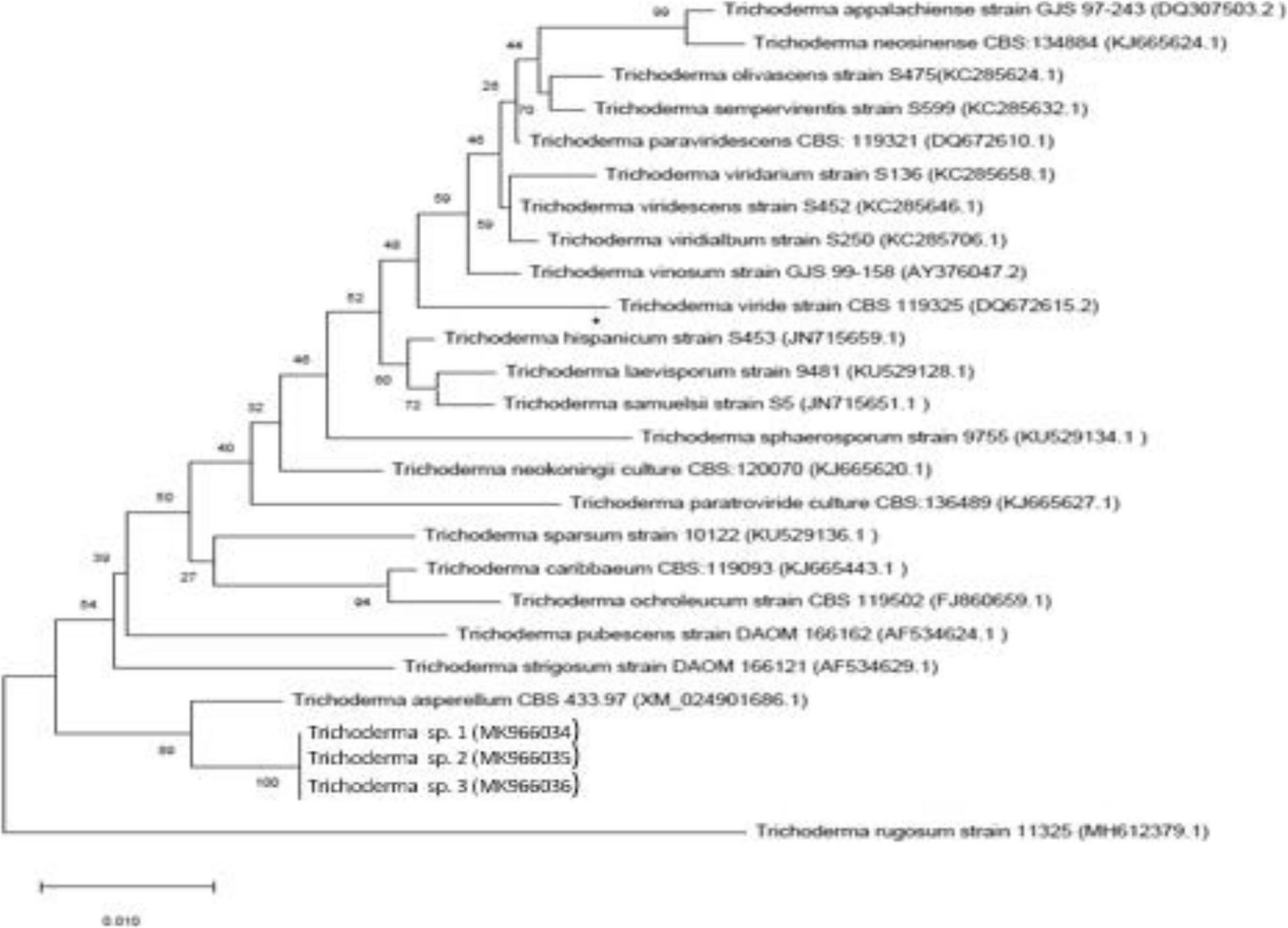
Phylogenetic reconstruction for the strains *Trichoderma* sp. 1, 2 and 3 based on elongation factor 1-alpha (EF1a) analysis using the neighbor-joining algorithm (NJ) method and 1000 replicate bootstraps (MK966034, MK966035 and MK966036 are the accession numbers of *Trichoderma* 1, 2 and 3 respectively)

Cultures of the pure isolates were run for molecular analysis with primers directed against the DNA sequences of the ITS region. Phylogenetic trees based on ITS sequences were constructed to find the relationships of the newly isolated strains to previously characterized species (Figs. 1 and 2). As shown in Fig. 1, phylogenetic analysis using ITS-derived sequences shows that our isolate, *Stemphylium* sp., clustered closely with *Stemphylium vesicarium* and *S. lucomagnoense*. In order to affiliate our isolate to one of these strains, morphological traits of the fungus were determined. After 3 weeks on MEA at 25 °C, colony reached 4–5 cm diameter. The white aerial mycelium became pale olivaceous grey at margin, producing flexuous, unbranched, smooth, hyaline to pale yellowish brown conidiophores (28–)35–85 × 3–4 μm, with conidiogenous cells enlarged at apex, pale brownish, 5–7 μm wide (Fig. 1). Conidia are solitary, ellipsoid, dark brown, and verrucose (22–30 × 12–16 μm), with (1–2–)3 transverse septa and 1(− 2) longitudinal septa. As the morphological features correspond to those described by Woudenberg et al. [18], we affiliated this isolate to *S. lucomagnoense*. Based on phylogenetic analysis, we affiliated our second isolate to *A. nidulans* (Fig. 2). The sequences obtained were deposited at Genbank under accession numbers MK691703 and MK691704 for *S. lucomagnoense* and *A. nidulans* respectively. Three other strains were affiliated to the genus *Trichoderma* based on sequences of the TEF-1α region (Fig. 3). The three isolates clustered in a clade comprising exclusively 23 *Trichoderma* species, with high bootstrap values for each branch (Fig. 3). The related sequences, corresponding to strains *Trichoderma* sp. 1, *Trichoderma* sp. 2 and *Trichoderma* sp. 3 were deposited under accession numbers MK966034, MK966035 and MK966036, respectively. It can be inferred from the phylogenetic tree that the strain closest to isolates *Trichoderma* sp. 1, *Trichoderma* sp. 2 and *Trichoderma* sp. 3 is the species *Trichoderma asperellum.*

### Production of fungal culture supernatants with laccase-like activity

**Fig. 4:**
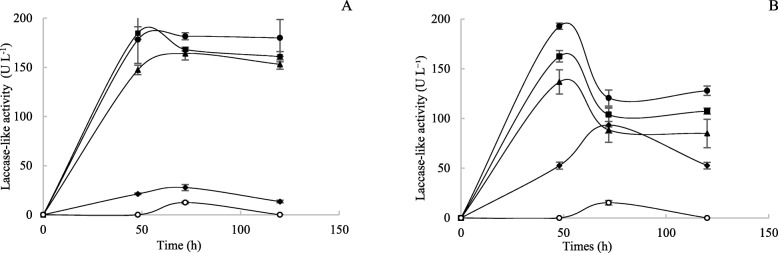
**a** Laccase activity of *Trichoderma asperellum* 1 (●), *Trichoderma asperellum* 2 (■), *Trichoderma asperellum* 3 (▲), *Stemphylium lucomagnoense* (♦) and *Aspergillus nidulans* (○) during 5 days of culture with ABTS as the substrate at pH 5.5 without (**a**) or with (**b**) 1% NaCl. Each data point (mean +/− standard deviation) is the result of triplicate experiments

Laccase-like activities of the five selected isolates were studied starting from liquid cultures. First we confirmed that the activity was not related to heme-containing peroxidase activities by adding either H_2_O_2_ or catalase to the reaction assay. Under these two conditions, no change in the activity was observed, suggesting that the activity is therefore not related to peroxidases (H_2_O_2_ dependent oxidases) but most probably correlated to laccases. Marked laccase-like activities were measured with *T. asperellum* 1, 2 and 3. *A. nidulans* and *S. lucomagnoense* produced lower activity levels. The highest laccase-like activities were detected with *T. asperellum* 1 and 2, with 185 U L^− 1^ (Fig. 4a). Laccase-like activities increased during the first 48 h and then reached a plateau. Because the five selected strains were isolated from marine environments, we assumed they were biologically adapted to living in saline conditions. We therefore tested whether the levels of secreted laccase-like activities were affected by adding 1% NaCl to the culture media (Fig. 4b). *T. asperellum* 1 yielded the highest level of laccase-like activities (193 U L^− 1^). For the three *T. asperellum* strains, secreted laccase-like activity sharply decreased after 48 h to level off at around 120 U L^− 1^, less than in cultures without NaCl, suggesting that the enzymes responsible might be sensitive to NaCl. Interestingly, laccase-like activity was significantly induced by adding NaCl to *S. lucomagnoense* cultures, yielding 110 U L^− 1^ (4–5 times more than the 25 U L^− 1^ obtained without NaCl). Because of the high levels of laccase-like activity in its culture supernatant, *T. asperellum* 1 was chosen for further studies.

### Effect of sea salt and different concentrations of NaCl on laccase-like activities in *Trichoderma asperellum* 1 cultures

**Fig. 5:**
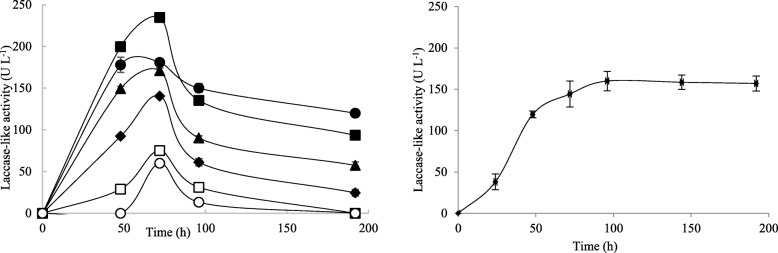
**a** Effect of different concentrations of NaCl (0% (●), 1% (■), 2% (▲), 3% (♦), 4% (□) and 5% (○)) on *Trichoderma asperellum* 1 laccase-like activity. **b** Effect of 1% of sea salt on *T. asperellum* 1 laccase-like activity. Each data point (mean +/− standard deviation) is the result of triplicate experiments

Different concentrations of NaCl (0, 1, 2, 3, 4 and 5% w/v) were added to the medium used for *T. asperellum* 1 cultures, and laccase-like activity in the resulting supernatant was quantified. The results are shown in Fig. 5a. As previously observed, the addition of 1% NaCl induced the production of laccase-like activities, with an optimum of 235 U L^− 1^ after 3 days of fungal culture. Laccase-like activity instead decreased at higher concentrations of NaCl. Natural seawater does not contain only sodium chloride, but also large quantities of chlorides and sulfates of calcium, potassium, and magnesium, and much lower amounts of many trace elements. Addition of 1% sea salt to *T. asperellum* 1 cultures was therefore also tested (Fig. 5b). In these conditions, no real effect on laccase-like activity was found, with a 160 U L^− 1^ maximum at Day 4, against 170 U L^− 1^ at Day 3 with no NaCl. Interestingly however, with sea salt, laccase-like activity did not decrease after 72 h, remaining stable up to 200 h growth.

### Influence of CuSO_4_ and of different carbon sources on laccase-like activity in *Trichoderma asperellum* 1

**Fig. 6:**
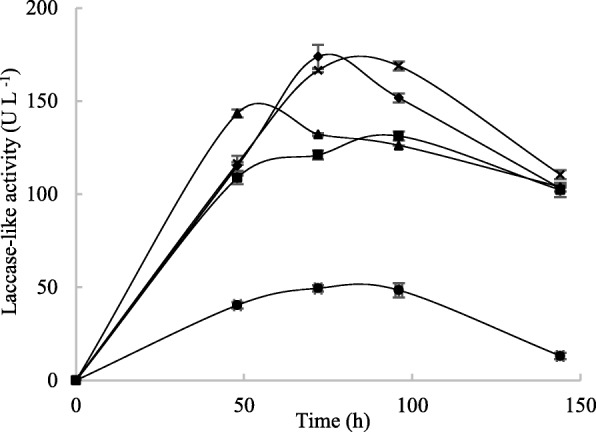
Effect of different concentrations of CuSO_4_ (0 mM (●), 0.8 mM (■), 1 mM (▲), 1.8 mM (♦) and 2 mM (ӿ)) on *Trichoderma asperellum* 1 laccase-like activity

To study the effect of CuSO_4_ on secreted laccase-like activity, different concentrations of CuSO_4_ (800 μM, 1000 μM, 1800 μM and 2000 μM) were added to the M7 medium used for *T. asperellum* 1 cultures. The results reported in Fig. 6 indicate that laccase-like activity increased significantly in the supernatant when cultures were supplemented with CuSO_4_. These increments were dose-dependent and significantly higher at around 2000 μM CuSO_4_, as clearly visible at 72 h, when activity (170 U L^− 1^) was more than 3 times higher than in cultures without CuSO_4_ (50 U L^− 1^).

Carbon sources are also known to strongly affect the levels of secreted fungal laccase-like activities. Accordingly, we tested the effect of adding 3% of sucrose, glucose or starch to the M7 production medium (Fig. S1 Supplementary data). We found that 3% sucrose resulted in higher levels of laccase-like activity (270 U L^− 1^) in the resulting supernatant.

### Decolorization of synthetic dyes

**Fig. 7:**
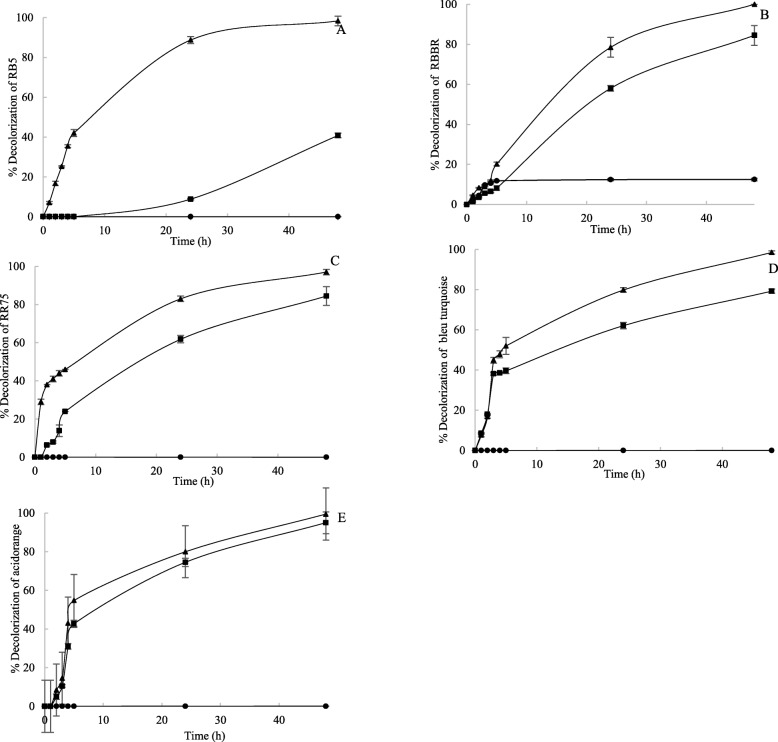
Decolorization of the five reactive dyes (50 mg L^− 1^ each), namely industry Reactive Black 5 (RB5) (**a**), Remazol Brilliant Blue R (RBBR) (**b**), RR75 (**c**), Blue Turquoise (**d**) and Acid Orange (E) in 48 h (% of decolorization in the presence of 1-hydroxybenzotriazole (HBT) (●), % of decolorization in the presence of enzyme (■) and % of decolorization in the presence of enzyme and HBT (▲)). The disappearance of the color by *Trichoderma asperellum* 1 culture supernatant was monitored at specific wavelengths (585, 597, 520, 438 and 606 nm) with time (1, 2, 3, 4, 5, 24 and 48 h). Each data point (mean +/− standard deviation) is the result of duplicate experiments

*T. asperellum* 1 cell-free supernatant was prepared in the optimized production medium (M7 containing 1% NaCl, 3% sucrose and 1.8 mM CuSO_4_). The decolorization ability of the culture supernatant was tested on five different dyes, belonging to three different dye families (reactive, azo and anthraquinone). The culture supernatant was incubated in the presence of five dyes (50 μg mL^− 1^ each), namely Remazol Brilliant Blue R (RBBR), Reactive Black 5 (RB5), Direct Red 75 (DR75), Acid Orange 51 (AO51) and Turquoise Blue (TB) for 48 h. Results showed that the presence of HBT, as observed for most laccases, improved the decolorization process, probably by facilitating electron transfer between oxidative enzymes from the culture supernatant and the substrate dye molecules. Figure 7 shows that in all cases HBT improved the decolorization efficiency of the *T. asperellum* 1 culture supernatant, but only with RB5 was it necessary. RB5 was barely decolorized with no mediator (only 9% decolorization), whereas in 24 h after addition of HBT the decolorization increased from 9 to 90%. With RBBR, DR75 and TB, the decolorization increased with the use of HBT from 60 to 80%, while for AO51 only 5% of additional decolorization was achieved (from 75 to 80%). Finally, our study shows that as observed for laccases, the addition of HBT enhances decolorization to different extents depending on the dye to be oxidized.

## Discussion

Fungi are recognized for their ability to produce a broad variety of extra-cellular enzymes [19]. However, most fungi studied to date have been isolated from forests and other terrestrial environments, and very few studies have explored marine fungal diversity. A large proportion of the diversity of marine-derived fungi may have originated from their terrestrial counterparts, with the appearance of strains able to live in harsh marine environments (high pressure, low temperature, oligotrophic nutrients, high salinity, etc.) [20, 21]. These specific conditions account for the significant differences between the enzymes generated by marine-derived microorganisms and their homologs from terrestrial counterparts [22]. Finally, marine-derived microorganisms have been studied to exploit their potential to generate new natural products and to degrade plant biomass [23].

In this study, 20 marine-derived fungi were isolated from Tunisian marine biotopes. Five of them were selected for their oxidative profile on DMP and ABTS. These five strains were identified as ascomycetes belonging to the species *Aspergillus nidulans*, *Stemphylium lucomagnoense* and *Trichoderma asperellum* (three strains belonging to the latter species). Among these marine-derived strains, *Aspergillus nidulans*, an anamorph of *Emericella nidulans,* is an important model ascomycete for eukaryotic genetics. A few studies have been dedicated to marine-derived *A. nidulans* species, such as two relatively recent ones reporting on the production of molecules of interest: melanin precursors with UVB protective properties [24] and antitumor alkaloids [25]. Another strain identified in this study belongs to the phylum ascomycetes (*Dothideomycetes, Pleosporales*, *Pleosporaceae*), specifically to the *Stemphylium* genus, that encompasses worldwide-distributed saprophytes and plant pathogens affecting a variety of agricultural crops. Molecular analysis branched *Stemphylium* sp. with both *S. vesicarium* and *Stemphylium lucomagnoense* in the phylogenetic tree, but morphological traits confirmed that the isolated species is *S. lucomagnoense*, an anamorph of *Pleospora lucomagnoense*. To date, only two studies have focused on marine-derived *Pleospora*. The first deals with the production of antimicrobial compounds [26] and the second with the phylogeny of *Pleospora gaudefroyi* [27].

A number of molecular markers have successfully been used for the taxonomic identification of fungal genera and species, and the ITS rDNA region has often been considered a marker of choice for the fungal kingdom [28]. However, sequencing of the TEF-1α region is considered a sensitive tool for identification in mycology, with better resolution than ITS, e.g. when studying the genus *Trichoderma* [29]. In this study, TEF-1α sequence-based phylogeny suggests that the species phylogenetically closest to our three isolates *Trichoderma* sp. 1, 2 and 3 is *Trichoderma asperellum*, a fungus naturally found in soils [30]. Although *Trichoderma* species are usually found in terrestrial habitats, some isolates have been collected from marine environments, where they live in association with algae [31] and sponges [32], in coastal sediments [33], or as endophytes in mangroves [34]. Among these marine-derived species we found *T. asperellum*, which was further studied for its production of secondary metabolites, such as sesquiterpenes [35] and antibacterial peptides [36].

Different *Trichoderma* species have been extensively studied as sources of cellulases, but also oxidases such as laccases [37]. This was the case, for instance, with the terrestrial species *Trichoderma reesei* [37]*, T. harzianum* and *T. longibrachiatum* [38], and for the marine-derived *Trichoderma* sp. [39]. A terrestrial *T. asperellum* producing oxidases including laccases was applied to degrade polycyclic aromatic hydrocarbons in soil [40]. In our study, the culture supernatant of five fungal isolates showed different amounts of laccase-like activities in liquid cultures and under saline conditions. The highest laccase-like activity was observed with the strain *T. asperellum* 1, in cultures with or without 1% NaCl. For comparison, while marine-derived *A. sclerotiorum* produced 9.26 U L^− 1^ laccase-like activity after 7 days culture in 3% (w/v) NaCl, *T. asperellum* 1 produced about 190 U L^− 1^. In another study [41], optimization of laccase-like activity levels from *Trichoderma* sp. grown in 0.5% NaCl yielded approximately 2000 U L^− 1^, but activity was assayed using *o*-tolidine instead of ABTS as a substrate, so that these results are not directly comparable with ours. The finding of laccase-like activities from fungal cultures grown in NaCl-containing media could be of benefit to industrial and biotechnological processes in which salinity is high [42]. In our study, we show that high levels of salt-tolerant laccase-like activity can be found using synthetic dyes as substrates. These findings open the way to the discovery of novel biocatalysts for the textile industry, whose effluents contain not only dyes, but also high salt concentrations. Secretome and enzyme characterization will be the next step in our research.

To maximize the levels of laccase-like activity in *T. asperellum* 1 cultures, we evaluated the effect of different concentrations of NaCl and known inducers, such as CuSO_4_ and three carbon sources. These parameters can affect the productivity of various oxidases secreted in the culture medium, owing to an inhibition of fungal growth or to effects on enzyme stability and activity, possibly in relation to protein surface charges and perturbation of global or local protein folding [43]. In our study, higher levels of laccase-like secreted activity were found when 1% NaCl was added to *T. asperellum* 1 cultures. Above this concentration, activity gradually decreased with increasing NaCl concentration. The effect of NaCl was also studied for other marine fungi such as *Cerrena unicolor* isolated from mangroves [44], and was shown to enhance laccase activity in fungal culture supernatants. Similarly, by adding sea salt to *T. asperellum* 1 cultures, we obtained an increase in the supernatant oxidase activity in time, with a maximum at 75 h (like with NaCl), but no decrease afterwards (unlike with NaCl). In previous studies we demonstrated the activation by sea salt of two laccases from the mangrove fungus *Pestalotiopsis* sp. [45], while a laccase from *Trematosphaeria mangrovei* lost 50% of its activity in 1% NaCl [14]. Salt-adapted enzymes are generally characterized by highly negative surface charges that are assumed to contribute to protein stability in extreme osmolytic conditions [46]. Copper has been reported to be a strong laccase inducer in several fungal species [47, 48]. It has been also reported that the increase in activity is proportional to the amount of copper added [49]. In our study, optimal CuSO_4_ concentration was 1.8 mM for *T. asperellum* 1 cultures, yielding about 173 U L^− 1^ laccase-like activity. These results are in agreement with previous ones [50], showing optimum laccase activity (32.7 U mL^−l^) in *Pestalotiopsis* sp. cultures with 2.0 mM CuSO_4_, and decreased activity above this concentration. Nakade et al. [51] reported that the best CuSO_4_ concentration for laccase production in *Polyporus brumalis* was 0.25 mM. CuSO_4_ induction of laccase is related to the active site architecture of these enzymes, which generally contain four copper atoms per polypeptide. Copper addition to the culture medium was also reported to induce laccase gene transcription [52]. In addition, it has been reported that copper can be toxic, as it interacts with nucleic acids, proteins, enzymes and metabolites associated with major cell functions, so that CuSO_4_ concentration should be checked case by case [52]. Several studies have proved that the choice of carbon sources affects the production of ligninolytic enzymes [53]. The purpose of glucose supplementation to lignocellulose for fungal cultures is twofold. First, it promotes the growth and rapid establishment of the fungus within the solid raw material. Second, the fungus needs an additional, easily metabolizable carbon source to sustain lignin degradation from lignocellulosic substrates [54]. In our study, sucrose was the best substrate for secreted laccase-like activity from *T. asperellum* 1 cultures (290 U L^− 1^), as previously shown for *Arthrospira maxima* [55].

Industrial dyes are usually of synthetic origin and have complex aromatic structures that make them highly resilient and more difficult to biodegrade [56]. Reactive dyes, for example, contain chromophore groups such as azo or anthraquinone. Most of these dyes are not toxic themselves, but after release into aquatic environments may be converted into potentially carcinogenic amines that impact the ecosystem downstream of the mill [57]. Currently employed physical and chemical methods have been shown to have some serious limitations, such as high cost, high salt content utilization, and problems related to the disposal of concentrate [58, 59]. In this regard, emphasis has been placed on developing biological processes, because they are more effective than more conventional, physical and chemical methods [57]. The production of oxidases with laccases from marine-derived ascomycetes, zygomycetes and basidiomycetes has been under-researched [42, 60]. Similarly, to our knowledge, only one study reports on the application of laccase-active supernatants from a marine *Trichoderma* to degrade synthetic dyes [41], one describes the production of laccase from marine-derived *Aspergillus sclerotiorum* [60] and no work is available on laccases derived from *Stemphylium* species*.* In this study, the dye decolorization ability of *T. asperellum* 1 culture supernatant was tested against five different industrial synthetic dyes: Reactive Black 5 (RB5), Remazol Brilliant Blue R (RBBR), Direct Red 75 (DR75), Turquoise Blue (TB) and Acid Orange 51 (AO51). These dyes belong to different dye families: reactive, azo and anthraquinone. It is generally observed that the extent of decolorization depends on the enzyme properties (and so the biological source) together with the chemical properties, structure and size of the dye molecule [2, 61]. Owing to their high molecular weight, for example, sulfonated azo dyes are unable to pass through the cell membrane, and degradation of these dyes must therefore take place extracellularly. The role of redox mediators in azo bond detoxification has also already been shown [62]. For instance, it has been reported that adding the mediator HBT to the laccase-active culture supernatant of *Paraconiothyrium variabile* enhances the decolorization of RB5, RBBR, DR75 and TB [63].

In a previous study we investigated RBBR decolorization by the culture filtrate of the terrestrial ascomycete *Trametes trogii* and by a laccase isolated from it [64]. The purified laccase decolorized up to 97% of a 100 mg L^− 1^ dye solution, with only 0.2 U mL^− 1^ enzyme. In our test conditions, we reached comparable results (60–80% decolorization) with *T. asperellum* 1 culture supernatant, with or without HBT. In general, different marine-derived strains will degrade RBBR to different extents, for example *Flavodon flavis* degraded RBBR by more than 90% [65], but *Cerrena unicolor* only by 46% [66].

Biodegradation of RB5 was investigated using the culture supernatant of the *Trichoderma atroviride* F03 yielding 91.1% decolorization without mediators [67]. Three products of this biodegradation reaction (1, 2, 4-trimethyl benzene, 2, 4-ditert butylphenol and benzoic acid-TMS derivatives) were identified, confirming the validity of enzymatic treatment without generating aromatic amines, which are highly toxic [67]. In comparison, the *T. asperellum* 1 culture supernatant achieved only 10% of RB5 decolorization without HBT, and up to 80% in the presence of the mediator.

AO51 is a water-soluble anionic azo dye. Typically containing one to three sulfonic groups, it is widely applied to color wool, silk and polyamide. The nature and level of toxicity of AO51 has not yet been well established [68], but sulfonated azo dyes (including naphthalene sulfonic acids, naphthols, naphthoic acids, benzidines, etc.), and particularly benzidines are a focus of attention because of their carcinogenicity [68]. AO51 degradation by crude laccase from *Trametes trogii* grown in solid cultures on sawdust has been investigated [68], and above 88% decolorization in the presence of HBT was achieved. Our results show that by contrast, with *T. asperellum* 1 culture supernatant, HBT was not essential for AO51 decolorization. To our knowledge, this is the first report of AO51 decolorization with no need for laccase mediators.

To date, only a few studies have dealt with decolorization of the phthallocinine dye TB. Plácido et al. showed that *Leptosphaerulina* sp. effectively decolorized TB and two real effluents from textile industries [69]. This decolorization was catalyzed by the production of significant quantities of laccase (650 U L^− 1^) and manganese peroxidase (100 U L^− 1^). *Leptosphaerulina* sp. enzymatic extracts exhibited decolorizing activity when ABTS was added as a mediator. Similarly, the culture supernatant of *T. asperellum* 1 showed maximum TB biodegradation capacity when HBT was added.

Remarkably high levels of DR75 degradation (95–100%) were achieved after 120 h incubation with *Penicillium oxalicaum* culture supernatant [70]. In that study, high levels of manganese peroxidase activity (659.4 ± 20 U L^− 1^) were measured in the culture supernatant of *P. oxalicaum*, indicating the involvement of heme peroxidases in the decolorization process. By contrast, in our study no peroxidase activity was detected in the culture supernatant of *T. asperellum* 1, suggesting for the first time to our knowledge that oxidase-catalyzed DR75 degradation takes place instead.

Further studies will be needed to gain further insight into the enzymatic mechanisms deployed by marine-derived fungi to cope with their environment. It will be necessary to identify the key enzymes secreted by *T. asperellum* 1 growing in saline conditions, and to produce and characterize them, with a focus on salt-dependency and the structure-function relationship underlying enzyme properties. To assess the potential of the culture supernatant of *T. asperellum* 1 or enzymes for enzymatic bioremediation of textile effluents, the degradation products of enzymatically treated model dyes and industrial samples need to be precisely identified and characterized, and their impact on human health and environment determined.

## Conclusion

In this work, we collected several fungal samples from the harbour of Sfax, Tunisia. After a purification procedure, the molecular and morphological identification of these samples showed that the isolate fungal strains correspond to *Trichoderma asperellum, Stemphylium lucomagnoense* and *Aspergillus nidulans*. Analyzing their oxidase activities *T. asperellum* strain (*T. asperellum* 1) gave the highest level of secreted oxidative activities. Therefore, this study showed that the optimal growth conditions to produce laccase-like active cell-free supernatants from *T. asperellum* 1 were 1.8 mM CuSO_4_ as an inducer, 1% NaCl to mimic a seawater environment and 3% sucrose as a carbon source and the culture supernatant of this strain effectively decolorized different synthetic dyes belonging to diverse chemical classes, and the presence of HBT as a mediator improved the decolorization process.

## Methods

### Sample collection

The environmental samples (woods immersed in seawater, seaweeds, marine plants, pieces of nets) used in this study were collected from four different Tunisian marine biotopes: the fishing port, the Sidi Mansour and the Casino sites at Sfax, and the polluted Khnis site at Monastir. These sites were chosen because of their pollution, with the intention of isolating fungal strains resistant to polluted water, and enzymes able to work in the presence of several contaminant species and aromatic compounds. The samples were collected in sterile tubes using a sterile spatula and stored at 4 °C until use.

### Isolation of fungi

Small pieces of sample were inoculated on 3.9% (w/v) potato dextrose agar (PDA) (Sigma-Aldrich, Saint-Quentin-Fallavier, France) and 1.8% (w/v) malt extract (Sigma-Aldrich), with 3.4% (w/v) NaCl and 0.1% (w/v) chloramphenicol to prevent bacterial growth, and incubated at 30 °C for 3 days until fungal growth was observed. Apparently monomorphic cultures obtained after at least two transfers onto fresh agar plates were further authenticated using molecular tools to check strain purity and identity.

### Preliminary screening of the isolates

Preliminary screening for oxidative activity was performed in PDA plates supplemented with 2 mM 2,6-dimethoxyphenol (DMP) or 200 μM 2,2′-azino-bis-(3-ethylbenzthiazoline-6-sulfonic acid) (ABTS) as substrates. The plates were incubated at 30 °C for 3 days and the presence of orange and purple halos around the mycelium was considered as the positive sign of substrate oxidation.

### Molecular identification (DNA extraction, PCR and sequencing)

The mycelium of selected strains was obtained by liquid culture in 50 mL flasks in malt extract medium for 3 days. Genomic DNA was isolated from 40 to 80 mg of mycelium powder using a GeneJET Genomic DNA Purification Kit (Thermo Scientific, Waltham, USA) following the manufacturer’s instruction. DNA concentration was estimated at 260 nm using a Nanodrop 2000 instrument (Thermo Fisher Scientific, Wilmington, USA).

The extracted DNA was used as the template in a PCR to amplify the partial sequences of two DNA loci, namely the internal transcribed spacer region (ITS) and the translation elongation factor 1α region (TEF-1α). The primers used for the amplification were ITS5 (5′- GGAAGTAAAAGTCGTAACAAGG-3′) and ITS4 (5′-TCCT-CCGCTTATTGATATGC-3′) [71] for the former (used for the *Aspergillus* and *Stemphylium* isolates), and TEF1α-983-F-CF2 (5′-GCYCCYGGHCAYCGTGAYTTYAT-3′) and TEF1α-2218-R-CR2 (5′-ATGACACCRACRGCRACRGTYTG-3′) [29] for the latter (used for the *Trichoderma* strains). PCR was performed using a Expand High Fidelity Kit (Roche Diagnostics GmbH, Mannheim, Germany) in 5 μL buffer (100 mM Tris HCl, 150 mM MgCl_2_ and 500 mM KCl) with 1.5 mM MgCl_2_, 0.25 μM of each primer, 1 μL of deoxynucleoside triphosphate (200 μM of each dNTP), 1 μL of DNA (about 100 ng), and Taq DNA polymerase (25 mU. μL^− 1^), in a final volume of 50 μL. Cycling parameters were 94 °C for 2 min followed by 40 cycles of 94 °C for 15 s, 51 °C for 30 s, and 72 °C for 1 min, with a final extension at 72 °C for 10 min. Negative control reactions lacking template DNA were performed in parallel. Amplified fragments were visualized on 1% agarose gels (FlashGel™ System) and sequenced using the two PCR primers (Roche Diagnostics GmbH, Mannheim, Germany).

To deduce the phylogeny of the fungal isolates, the sequences ITS and TEF-1α were compared with data available at the public database Genbank by using the BLASTn sequence match algorithm [71]. The best hits for each species retrieved from the BLAST search were retained and used to construct phylogenetic trees. Sequences were aligned using the CLUSTAL W program [72], and phylogenetic and molecular evolutionary analyses were performed using MEGA X [73]. The phylogenetic tree was constructed using the neighbor-joining algorithm [74] with bootstrap values calculated from 1000 replicates [60].

The fungal strains were deposited at the Spanish type culture collection (CECT) under the reference numbers CECT 21166, CECT 21167 and CECT 21168 for *Trichoderma asperellum* 1, 2 and 3 respectively, CECT 21164 for *Stemphylium lucomagnoense* and CECT 21165 for *Aspergillus nidulans*.

### Fungal cultures

For solid late culture, *S. lucomagnoense* was grown on MEA (30 g of malt extract with 20 g of agar). For other fungal cultures, elected marine fungal strains were grown in submerged cultures in 50 mL M7 medium, and culture supernatant was used to retrieve ABTS-oxidizing laccase-like activity as previously described [75]. Fifty milliliter of 3-day precultures of fungal mycelia were vortexed using glass beads (0.6 mm) for 1 min. The homogenized mycelial fragments were used to inoculate 250 mL Erlenmeyer flasks containing 50 mL of M7 medium. The medium (M7) contained (g L^− 1^): glucose 5, peptone 5, yeast extract 1, ammonium tartrate 2, KH_2_PO_4_ 1, MgSO_4_.7H_2_O 0.5, KCl 0.5, trace element solution 1 mL. The trace element solution composition was (g L^− 1^): B_4_O_7_Na_2_.10H_2_O 0.1, CuSO_4_.5H_2_O 0.01, FeSO_4_.7H_2_O 0.05, MnSO_4_.7H_2_O 0.01, ZnSO_4_.7H_2_O 0.07, (NH_4_)_6_Mo_7_O_24_.4H_2_O 0.01. The final pH was adjusted to 5.5. The cultures were incubated at 30 °C and 160 rpm, and aliquots were withdrawn daily. Cu^2+^ induction was performed in M7 medium supplemented with 2 mM CuSO_4_.

### Laccase-like activity assay

Laccase-like activity was measured by monitoring the oxidation of 5 mM ABTS (Sigma-Aldrich) in 0.1 M citrate phosphate buffer (pH 5) at 436 nm for 1 min [76]. The reaction mixture (1 mL) contained 0.1 mL supernatant of the culture medium, which was centrifuged for 10 min at 12000 rpm. Oxidase activity was determined as the increase in absorbance at 436 nm [(*ε*_436nm_ = 29,300 M^− 1^ cm^− 1^) [77]. One unit of ABTS-oxidizing activity is defined as the amount of enzyme needed to oxidize 1 μmol of ABTS per minute at room temperature. Measurements were also conducted in the presence of either H_2_O_2_ (0.5 mM) or catalase (280 units per ml of assay) to confirm that no activity was due to heme-containing peroxidases.

### Influence of NaCl, sea salt, CuSO_4_ and different carbon sources on laccase-like activity

To compare the effect of NaCl and sea salt on the production of active cell-free supernatants, standard M7 medium was supplemented with increasing concentrations of either NaCl or sea salt (1–5% w/v). Fifty milliliter cultures were grown in 250 mL Erlenmeyer flasks for 7 days at 30 °C, and samples were withdrawn periodically. CuSO_4_ was also supplemented to cultures as an inducer of laccase-like activity in case laccases were involved. To determine the suitable concentration of CuSO_4_ for an optimal production of laccase-like activities, the following concentrations of CuSO_4_ were tested: 800 μM, 1000 μM, 1800 μM and 2000 μM. To find the suitable carbon source for highest laccase-like activity in culture supernatants, the effect of different carbon sources, such as sucrose, glucose and starch was studied. The carbon sources were tested at a concentration of 3% in M7 production medium. The Erlenmeyer flasks (250 mL) containing 50 mL of the production medium were incubated at 30 °C for a period of 7 days.

### Dye decolorization by the culture supernatant of *Trichoderma asperellum* 1

To test the ability of *T. asperellum* 1 cultures to decolorize industrial dyes, five different dyes used in the textile industry were selected: Remazol Brilliant Blue R (RBBR), Reactive Black 5 (RB5), Direct Red 75 (DR75), Acid Orange 51 (AO51) and the Turquoise Blue (TB). Dyes were solubilized in water at a concentration of 500 mg L^− 1^. Each dye was incubated at 30 °C in 0.1 M phosphate-citrate buffer pH 5.0 at a final concentration of 50 mg L^− 1^, together with aliquots of culture supernatant accounting for total ABTS-oxidizing activity of 0.6 U L^− 1^, in a final volume of 1 mL. Measurements were conducted in the presence or absence of 1 mM 1-hydroxybenzotriazole (HBT). Color disappearance was monitored at the maximum absorbance wavelength for each dye (585, 597, 520, 438 and 606 nm for RBBR, RB5, DR75, AO51 and the TB respectively). For each reaction mixture, absorbance was recorded at 1, 2, 3, 4, 5, 24 and 48 h. The percentage decolorization was calculated by taking the maximum absorbance of each untreated dye solution as the control (100% color). Optical density was measured using an Optizen Pop QX UV/Vis spectrophotometer (Klab, King of Prussia, USA). All experiments were performed in triplicate.

Decolorization was defined as the percentage of absorbance loss compared to the control, untreated dye solution (defined as 100% absorbance, ABSORBANCE *t*_0_), using the formula:

$$ \mathrm{decolorization}\ \left(\%\right)=\frac{\left(\mathrm{ABSORBANCE}\ t0-\mathrm{ABSORBANCE}\ t\mathrm{f}\right)\times 100}{\mathrm{ABSORBANCE}t0.} $$

## Supplementary information

**Additional file 1 **: **Figure S1** Effect of different sources of carbon (glucose (●), sucrose (▲), starch (■)) on *Trichoderma asperellum* 1 laccase-like activity.

## Data Availability

All the data generated and analyzed during this study are included in the published article. The fungal strains were deposited at the Spanish type culture collection (CECT) under the reference numbers CECT 21166, CECT 21167 and CECT 21168 for *Trichoderma asperellum* 1, 2 and 3 respectively, CECT 21164 for *Stemphylium lucomagnoense* and CECT 21165 for *Aspergillus nidulans*.

## Abbreviations

HBT: 1-hydroxybenzotriazole
DMP: 2,6-dimethoxyphenol
ABTS: 2,2′-azino-bis-(3-ethylbenzthiazoline-6-sulfonic acid)
MEA: Malt extract agar
PDA: Potato dextrose agar
ITS: The internal transcribed spacer region
TEF-1α: Translation elongation factor1α region
RBBR: Remazol Brilliant Blue R
RB5: Reactive Black 5
DR75: Direct Red 75
AO51: Acid Orange 51
TB: Turquoise Blue
CECT: Spanish Type Culture Collection
H_2_O_2_: Hydrogen peroxide
h: hour
