# Supplementary figures and images for: Screening of five marine-derived fungal strains for their potential to produce oxidases with laccase activities suitable for biotechnological applications
Source: BMC Biotechnol. 2020 May 12;20:27. doi: 10.1186/s12896-020-00617-y (PMC7218534; doi:10.1186/s12896-020-00617-y)

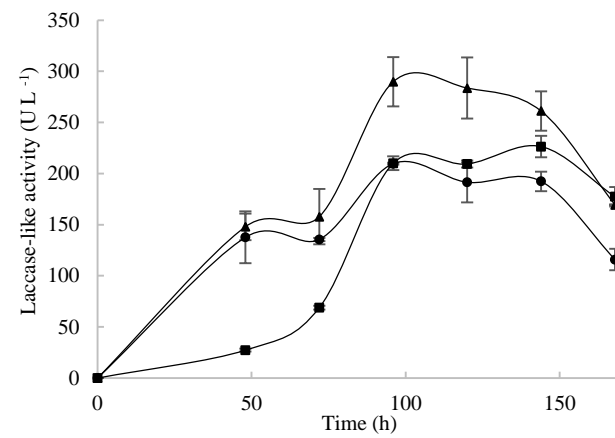

**Fig. 1S**

Supplement: Supplementary file 1 — Additional file 1 : Figure S1 Effect of different sources of carbon (glucose (●), sucrose (▲), starch (■)) on Trichoderma asperellum 1 laccase-like activity. [file 12896_2020_617_MOESM1_ESM.pdf]
